# Solvent Extraction of PDMS Tubing as a New Method for the Capture of Volatile Organic Compounds from Headspace

**DOI:** 10.1007/s10886-024-01469-y

**Published:** 2024-01-22

**Authors:** Gareth Thomas, John Caulfield, Lucia Nikolaeva-Reynolds, Michael A. Birkett, József Vuts

**Affiliations:** 1 Protecting Crops and the Environment, Rothamsted Research, Harpenden, AL5 2JQ UK; 2 School of Biological Sciences, University of Bristol, 24 Tyndall Avenue, Bristol, BS8 1TQ UK

**Keywords:** Headspace sampling, PDMS, Semiochemical, Volatile, Solvent extract, Silicon

## Abstract

Polydimethylsiloxane (PDMS) tubing is increasingly being used to collect volatile organic compounds (VOCs) from static biological headspace. However, analysis of VOCs collected using PDMS tubing often deploys thermal desorption, where samples are considered as ‘one-offs’ and cannot be used in multiple experiments. In this study, we developed a static headspace VOC collection method using PDMS tubing which is solvent-based, meaning that VOC extracts can be used multiple times and can be linked to biological activity. Using a synthetic blend containing a range of known semiochemicals (allyl isothiocyanate, (*Z*)-3-hexen-1-ol, 1-octen-3-one, nonanal, (*E*)-anethol, (*S*)-bornyl acetate, (*E*)-caryophyllene and pentadecane) with differing chemical and physicochemical properties, VOCs were collected in static headspace by exposure to PDMS tubing with differing doses, sampling times and lengths. In a second experiment, VOCs from oranges were collected using PDMS sampling of static headspace versus dynamic headspace collection. VOCs were eluted with diethyl ether and analysed using gas chromatography – flame ionization detector (GC-FID) and coupled GC – mass spectrometry. GC-FID analysis of collected samples showed that longer PDMS tubes captured significantly greater quantities of compounds than shorter tubes, and that sampling duration significantly altered the recovery of all tested compounds. Moreover, greater quantities of compounds were recovered from closed compared to open systems. Finally, analysis of orange headspace VOCs showed no qualitative differences in VOCs recovered compared to dynamic headspace collections, although quantities sampled using PDMS tubing were lower. In summary, extraction of PDMS tubing with diethyl ether solvent captures VOCs from the headspace of synthetic blends and biological samples, and the resulting extracts can be used for multiple experiments linking VOC content to biological activity.

**Supplementary Information:**

The online version contains supplementary material available at 10.1007/s10886-024-01469-y.

## Introduction

Novel chemistries that act via a non-toxic mode of action are required to underpin the development of sustainable and environmentally benign pest management tools for improved food production. Semiochemicals, e.g. pheromones, are naturally-occurring behaviour- and development-modifying chemical signals that govern intra- and interspecific ecological interactions (Regnier 1971; Calcagnile et al. 2019; Beck and Vannette 2017; Agelopoulos et al. 1999; Beck et al. 2017). Understanding the role of volatile organic compounds (VOCs) as semiochemicals in mediating such interactions requires application of techniques for collection and interrogation of headspace from a range of organisms (Alborn et al. 2021).

Methods commonly used to sample headspace VOCs are broadly categorised into dynamic and static collections (Alborn et al. 2021; Agelopoulos and Pickett 1998). Dynamic headspace collections involve the passage of purified air through enclosed systems (e.g. air entrainment, closed-loop stripping apparatus) and the adsorption of VOCs onto polymers (e.g. Porapak Q, Tenax, charcoal), which are subsequently eluted with a high-purity solvent (Porapak Q) or thermally desorbed (Tenax) prior to analysis (Brezolin et al. 2018; Barbosa-Cornelio et al. 2019; Birkett 2010). Resulting samples are typically analysed using high-resolution gas chromatography-flame ionization detector (GC-FID) and coupled GC-mass spectrometry (GC-MS). By contrast, static headspace collections do not require the dynamic passage of air through the system, and analytes accumulate passively onto a sorbent material exposed to the headspace of a sample within a closed system. One of the main techniques for static headspace collection is solid-phase microextraction (SPME), which involves a syringe containing a fused silica fibre coated with a sorbent phase. This is exposed to the headspace of a sample for a period of time, during which analytes absorb into the fibre coating. After sampling, compounds are thermally desorbed from the fibre during GC-FID/GC-MS analysis (Merkle et al. 2015; Augusto and Luiz Pires Valente 2002; Musteata and Pawliszyn 2007). SPME fibres are coated with adsorbent material, e.g. polydimethylsiloxane (PDMS), divinylbenzene, carboxen, polyacrylate or polyethylene glycol, each having an influence on the relative recovery of compounds (Noushini et al. 2020; Stoppacher et al. 2010; Smith et al. 2000; Zini et al. 2001). Stir-bar sorptive extraction (SBSE) (Rykowska and Wasiak 2013) consists of a magnetic stir bar encapsulated in a polymeric PDMS coat that accumulates VOCs over time, which partition into the vapour phase during thermal desorption GC-FID/GC-MS analysis. This technique has been used routinely to collect VOCs from the headspace of a range of plant species (Ulrich and Wijaya 2010; Carlomagno et al. 2016; Kfoury et al. 2017; Thompson et al. 2021), as well as to extract attractants from water samples for mosquitoes (*Culex quinquefasciatus*), where the VOCs were eluted from the stir bars with organic solvent and resultant extracts used for behavioural and electrophysiological tests (Carson et al. 2010).

PDMS is an apolar, sorptive polymer, which absorbs lipophilic compounds and is commonly used as the stationary phase in GC capillary columns, as well as for fibre coating in SPME. Sorptive extraction involves the partitioning of analytes between the headspace and PDMS phases in three steps: (i) sorption from the headspace onto the PDMS, (ii) diffusion through the PDMS layer and (iii) absorption onto the inner phase (Bicchi et al. 2002, 2005). Parameters which influence analyte recovery using PDMS from headspace include the amount of PDMS and stir bar contact surface, temperature, time, headspace volume and stir bar length (Bicchi et al. 2005). These factors should therefore be taken into consideration when designing headspace collection methods for the study of semiochemicals.

PDMS microtubing has been used to investigate plant VOC production in situ, as PDMS tubing selectively absorbs VOCs that dissolve into the tubing rather than binding to it (Baltussen et al. 1998, 2002). This sampling method is a variation on SPME, although PDMS tubing can supply an increased extractive phase volume relative to SPME. Moreover, unlike SPME, analytes which have absorbed into PDMS tubing can then be eluted by passing an organic solvent through the tubing, and a portion of the sample is analysed by GC-FID/GC-MS.Weidenhamer (2005) first investigated the efficacy of PDMS tubing for the capture of sorgoleone from the rhizosphere of sorghum (*Sorghum bicolor*) by extracting the tubing with methanol. Compared to SBSE and technical-grade optical fibre coated with PDMS, it retained the highest quantity of sorgoleone. Similarly, PDMS tubing has been used to sample thiophenes from the roots of African marigold (*Tagetes erecta*) in soil, again using methanol for extraction (Mohney et al. 2009). Diethyl ether extraction of PDMS tubing in a sterile sand environment successfully isolated (*E*)-caryophyllene from the rhizosphere of maize plants damaged by *Diabrotica v. virgifera* larvae via analysing extracts directly by GC/GC-MS (Vuts et al. 2020).

There is increased interest in using PDMS tubing for sampling headspace VOCs. Such studies have taken place across a range of organisms, including *Nicotiana attenuata* leaves and flowers (Kallenbach et al. 2014), webs of social spiders (*Stegodyphus dumicola*) (Lammers et al. 2021), bean plants (*Phaseolus vulgaris* L.) (Karamanoli et al. 2020), poplar trees (*Populus nigra*) (Fabisch et al. 2019), tomato (*Solanum lycopersicum*) (Kong et al. 2021), lima bean (*Phaseolus lunatus* L.) (Song et al. 2022a), and *Arabidopsis* inoculated with *Bacillus amyloliquefaciens* (Song et al. 2022b). Recent work suggests PDMS tubing can be used to detect changes in barley (*Hordeum vulgare*) volatiles after mechanical wounding (Laupheimer et al. 2023). In each of these examples, thermal desorption was used for analysis by placing the PDMS tubing directly into a GC desorption unit following sampling. The advantages of thermal desorption include (i) the absence of a solvent peak in the GC-FID/GC-MS analysis, making the detection of low molecular weight compounds possible, and (ii) increased sensitivity due to the temperature-dependent removal of analytes from the polymer (Gaffke and Alborn 2021). However, thermal desorption is a destructive analysis method, therefore no sample is available for further chemical or behavioural assays following chromatographic analysis. This could be overcome by using solvent elution, for example with diethyl ether, to generate liquid extracts (Vuts et al. 2020). Such extracts will allow multiple chemical (GC-FID, GC-MS, NMR) and biological analyses of the resulting samples, aiding absolute compound identifications and establishing biological activity, which is dependent on adequate concentration of the target analytes recovered in the extracts (Birkett 2010). Moreover, as the use of PDMS tubing for headspace sampling is a recently developed sampling method compared to dynamic headspace collections and SPME, various parameters which could influence compound recovery, including tube length and sampling duration, have not yet been evaluated.

The main aim of this study was to determine whether VOCs can be extracted by pushing a bolus of organic solvent through PDMS tubing exposed to biological headspace. Using synthetic VOC blends and oranges as the model biological material, we determined key sampling parameters. We show that extraction of PDMS tubing with diethyl ether captures VOCs from the headspace of synthetic blends and biological samples, and that these extracts can be used for multiple experiments, linking VOC content to biological activity. The advantages of solvent extracts are (i) they can be easily concentrated, (ii) can be analysed using GC-FID, coupled GC-MS, and coupled GC-electroantennography (GC-EAG), (iii) can be used to confirm the identity of tentatively identified compounds through GC co-injections, and (iv) can be used in insect behavioural bioassays, making it possible to link the chemistry of the solvent extracts to electrophysiological and behavioural activity.

.

## Methods

### Experiments with Synthetic Compounds

For VOC collection experiments using a synthetic blend of semiochemicals, components within the blend were chosen to represent a range of chemical classes, boiling points, molecular weights and polarities, which have also been reported to be produced from plants, insects and microbes and possess a range of semiochemical properties (Table 1). Analytical stock solutions of each compound were prepared and aliquoted to generate an eight-component synthetic blend.

Polydimethylsiloxane (PDMS) tubing (1 mm ID x 0.4 mm wall thickness) was obtained from VWR international Ltd (Lutterworth, UK). Prior to each experiment, PDMS tubing was cut into pieces (2.5–7.5 cm, depending on the experiment) and soaked in 100% methanol for up to 24 h (Vuts et al. 2020), then placed into a glass vessel under a constant flow of purified nitrogen for 1.5 h within a modified heating oven (180˚C).

Prior to each experiment, glass chambers (6 cm height × 12 cm diam.) were cleaned using Teepol reagent followed by acetone, then rinsed with distilled water and baked at 150˚C for a minimum of 2 h. For headspace experiments using PDMS tubing, a small piece of tin foil was placed over the top of the outlet of the glass chamber and gently pierced using forceps. PDMS tubing was then suspended in the headspace. Within the chamber, a filter paper disc (6 mm diam.) placed onto a plastic Petri dish (5 cm diam.) was used to dispense the synthetic blend (Supplementary Fig. 1). Whilst the system could not be described as entirely closed due to the hollow tube used, a preliminary experiment indicated that there was no difference in compound recovery when the hollow tube was left unsealed, compared to when the top of the tube was closed with a crocodile clip (Supplementary Fig. 2). Therefore, unsealed tubes were used for subsequent experiments. PDMS tubes were suspended in glass chambers containing filter paper with the synthetic blend of compounds. Ten µL of the eight-component synthetic blend was then applied to the filter paper in each experiment, equating to a 100 µg dose/component, unless otherwise stated, using redistilled diethyl ether as the carrier solvent.

To elute the compounds at the end of each experiment, PDMS tubes were extracted with diethyl ether (1 mL) by inserting the narrow end of a Pasteur pipette into one end of the tube and the other into a glass vial (1.1 mL, Thermo Fisher Scientific, Hemel Hempstead, UK), and administering diethyl ether into the Pasteur pipette. The remaining solvent was extruded using a bolus of air from the pipetting bulb (as described in Vuts et al. 2020). For experiment 1, PDMS tubes were either eluted using 1 mL diethyl ether or inserted individually into glass Tenax tubes and thermally desorbed (see sample analysis for gas chromatography conditions). For experiment 5, PDMS tubes were suspended above the blend enclosed in a glass chamber or suspended over a piece of filter paper in an open lab environment at 1 or 4 cm distances. When handling PDMS tubes, cotton blend glove liners (Sigma Aldrich, Gillingham, UK) were used to prevent skin contaminants absorbing into the PDMS tube.

**Table 1:** Synthetic compounds used in the PDMS experiments

Compound	Purity/supplier	Kováts index (on a HP-1 non-polar GC column)	Molecular weight	Octanol:water partition coefficient	Example of ecological function	Reference
Allyl isothiocyanate	95%, Sigma Aldrich	854	99	1.8	*Brassica* compound, demonstrating antifungal activity	(Mayton 1996)
(*Z*)-3-Hexen-1-ol	95%, Fluorochem	844	100	1.33	Green leaf volatile, with a range of biological activities	(Visser 1979)
1-Octen-3-one	96%, Sigma Aldrich	960	126	2.49	Fungal compound	(Chen and Wu 1984)
Nonanal	95%, Sigma Aldrich	1084	142	2.99	Bacterial compound, demonstrating antifungal activity	(Fernando et al. 2005)
(*E*)-Anethol	99%, Sigma Aldrich	1275	148	2.94	Insect attractant	(Tóth et al. 2003)
(*S*)-Bornyl acetate	99%, Sigma Aldrich	1265	196	2.35	Floral volatile, insect attractant	(Vuts et al. 2018)
(*E*)-Caryophyllene	98%, Sigma Aldrich	1429	204	4.52	Plant-derived compound, attracts natural enemies of root-feeding herbivores	(Rasmann et al. 2005)
Pentadecane	99%, Sigma Aldrich	1500	212	7.13	Bacterial compound, plant growth promoting	(Blom et al. 2011)

The following experiments were performed:

*Experiment 1: Effect of desorption method on recovery of synthetic compounds*:

Dose of synthetic blend: 100 µg. PDMS tube length: 5 cm. Sampling duration: 1 h. Number of PDMS tubes per replicate: 1. Number of replicates per treatment: 4.

*Experiment 2: Effect of dose of synthetic blend on recovery of compounds*:

Dose of synthetic blend: 1 µg, 10 or 100 µg. PDMS tube length: 5 cm. Sampling duration: 1 h. Number of PDMS tubes per replicate: 1. Number of replicates per treatment: 4.

*Experiment 3: Effect of sampling time on recovery of compounds*:

Dose of synthetic blend: 100 µg. PDMS tube length: 5 cm. Sampling duration: 1 h, 3 h, 6 or 18 h. Number of PDMS tubes per replicate: 1. Number of replicates per treatment: 4.

*Experiment 4: Effect of tube length on recovery of compounds*:

Dose of synthetic blend: 100 µg. PDMS tube length: 2.5 cm, 5 or 7.5 cm. Sampling duration: 1 h. Number of PDMS tubes per replicate: 1. Number of replicates per treatment: 4.

*Experiment 5: Effect of enclosing source of compounds on recovery*:

Dose of synthetic blend: 100 µg. PDMS tube length: 5 cm. Sampling duration: 1 h. Number of PDMS tubes per replicate: 1. Number of replicates per treatment: 4. Distance of PDMS tube from source: 1 or 4 cm.

A second dose-response experiment was established to determine the dose of compounds which could be recovered from an open system. Dose of synthetic blend: 100 µg, 10 µg and 1 µg. PDMS tube length: 5 cm. Sampling duration: 1 h. Distance of PDMS tube from source: 1 cm. Number of PDMS tubes per replicate: 1. Number of replicates per treatment: 4. PDMS tubes were suspended 1 cm above the filter paper with the blend. A summary of the experiments performed can be found in Table 2.

**Table 2:** Summary of experiments

Experiment number/Parameter being tested	Dose of synthetic blend (µg)	PDMS tube length (cm)	Sampling duration (h)
1. Desorption method	100	5	1
2. Dose of synthetic blend	1, 10 or 100	5	1
3. Sampling time	100	5	1, 3 or 6
4. PDMS tube length	100	2.5, 5 or 7.5	1
5. Enclosing source of compounds	100	5	1

*Experiment 6: Use of PDMS tubing to capture VOCs from the headspace of oranges*:

Oranges (*Citrus sinensis* L. cv Navel) were selected as the plant species used for VOC collections, as their VOCs have been previously identified at Rothamsted (Chamberlain et al. 2012; Fancelli et al. 2018). *C. sinensis* fruits were prepared as described previously in Chamberlain et al. (2012), with amendments. Oranges were first washed with water and allowed to dry overnight. On the day of the experiment, individual oranges were pierced with a fine needle 30 times, and one orange was placed into a clean glass chamber (12 cm diam. × 10 cm height). A 5 cm piece of PDMS tubing was suspended above the headspace of each orange, which were sampled for one hour. Two treatments were established: one within a glass chamber (enclosed) attached to a metal plate with bulldog clips, and one where PDMS tubes were suspended above oranges which were not confined within glass chambers (non-enclosed). Number of replicates per treatment: 4. Blank control samples were prepared containing a PDMS tube suspended above an empty glass chamber, a PDMS tube suspended in blank air in the same room as the experiment, and a PDMS tube suspended in blank air in a different room.

To compare the compounds being captured using PDMS tubing with well-established VOC collection methods, dynamic headspace collections (air entrainment) were performed. One orange was enclosed within a glass chamber (12 cm diam. × 10 cm height) attached to a metal plate with bulldog clips. Air was pumped through an activated charcoal filter at 600 mL min^− 1^ in each chamber to provide a positive pressure of clean air. A glass tube containing 50 mg Porapak Q adsorbent sealed between glass wool plugs was placed in the air outlet and air was drawn through at a flow rate of 500 mL min^− 1^ to ensure a positive pressure through the system. Collections were performed for 1 h and adsorbent tubes eluted using ca. 1 mL of freshly distilled diethyl ether. Extracts were stored at -20 °C until analysis. For samples collected via air entrainment, extracts were not concentrated down, as this turned out to overload the GC column. PDMS extracts were concentrated to 100 µL under a gentle stream of N_2_.

### Gas Chromatography Flame Ionization Detector (GC-FID) Analysis

Diethyl ether extracts eluted from PDMS tubes were analysed on an Agilent 6890 GC equipped with a cool on-column injector, a flame ionization detector (FID) and a HP-1 bonded-phase fused silica capillary column (50 m × 0.32 mm i. d. × 0.52 μm film thickness) (Agilent, Santa Clara, CA, USA). The oven temperature was set at 30 °C for 0.1 min, then increased at 5 °C/min to 150 °C for 0.1 min, then at 10 °C/min to 230 °C for a further 25 min. The carrier gas was hydrogen. Aliquots of the extracts (4 µL) were injected into the GC. Quantification of compounds was achieved using the single-point external standard method using a series of C_7_-C_22_ alkanes.

For samples which were analysed using thermal desorption, PDMS tubes (5 cm) were inserted into a hollow glass Tenax tube and placed directly into the OPTIC Programmable Temperature Vaporisor (PTV) unit (30 -> 250 °C ballistically at a rate of 16 °C/s) of a GC (see above for specifications). The oven temperature was set at 30 °C for 0.1 min, then increased at 5 °C/min to 150 °C for 0.1 min, then at 10 °C/min to 230 °C for a further 25 min. The carrier gas was hydrogen.

### Coupled Gas Chromatography-Mass Spectrometry (GC-MS) Analysis

Coupled GC-mass spectrometry (GC-MS) analysis of eluted VOCs was performed using an Agilent GC-Mass Selective Detector System (5977B inert plus, source temperature 220 °C) coupled with an Agilent GC (8890 GC) fitted with a HP-1 capillary column (50 m × 0.32 mm inner diameter, 0.52 μm film thickness). Injection of eluted VOC samples was via a cool-on-column injector, with helium as the carrier gas. The oven temperature was maintained at 30 °C for 1 min and increased at 5 °C/min to 150 °C, where it was held for 0.1 min, then at 10 °C/min to 230 °C and held for 26 min. Tentative identifications were made by comparison of mass spectra with NIST11 mass spectral database and by comparison of GC retention indices (Kováts Index, KI). Where commercial standards were available, tentative identifications by GC-MS was confirmed by peak enhancement on a HP-1 column by co-injection with authentic compounds, using an Agilent 7890 A GC equipped with a cool on-column injector, FID and a 50 m × 0.32 mm i.d. HP-1 column (0.52 μm film thickness).

### Extraction Recovery Calculations

Extraction yields were calculated as previously described (Vuts et al. 2020; ref 7), using the formula ƞ = 1/((β/K_ow_) + 1), where ƞ is the extraction yield (recovery), β is the phase ratio of the static extraction system and is defined as V_medium_/V_PDMS_, and K_ow_ is the octanol-water partition coefficient. β was calculated using the following parameters: V_medium_ = 660 mL, V_PDMS_ = [r (0.09 cm)^2^ x Π x length within headspace (5 cm)]-[r of internal hole (0.05 cm)^2^ x Π x length within headspace (5 cm)] = 0.088 mL. The octanol-water partition coefficient (log K_ow_) values for each of the compounds were extracted from CHEMBL database (www.ebi.ac.uk/chembl/): allyl isothiocyanate = 1.80; (*Z*)-3-hexen-1-ol = 1.33; 1-octen-3-one = 2.49; Nonanal = 2.99; (*E*)-anethol = 2.94; bornyl acetate = 2.35; (*E*)-caryophyllene = 4.52; pentadecane = 7.13.

### Statistics

A log-to-base 10 transformation was applied to the amount (ng) of compounds to ensure data conformed to statistical assumptions. To test the statistical difference of means between two treatments, a two-sample t-test was used (*p* < 0.05). For analysis of means across three or more treatments, ANOVA, providing an F-test for the overall difference between treatments, was used, followed by application of Tukey’s post-hoc test (*p* < 0.05). The Genstat (2022, 21st edition, VSN International 140 Ltd, Hemel Hempstead, UK) was used for statistical analysis.

### Chemicals

Diethyl ether (99.5%) was purchased from Fisher Scientific, UK. Allyl isothiocyanate (95%), (Z)-3-hexen-1-ol (98%), 1-octen-3-one (96%), nonanal (95%), (E)-anethol (99%), (S)-bornyl acetate (95%), (E)-caryophyllene (98%), pentadecane (>99%) were from Sigma-Aldrich, UK.

## Results

### Experiment 1

**Fig. 1:**
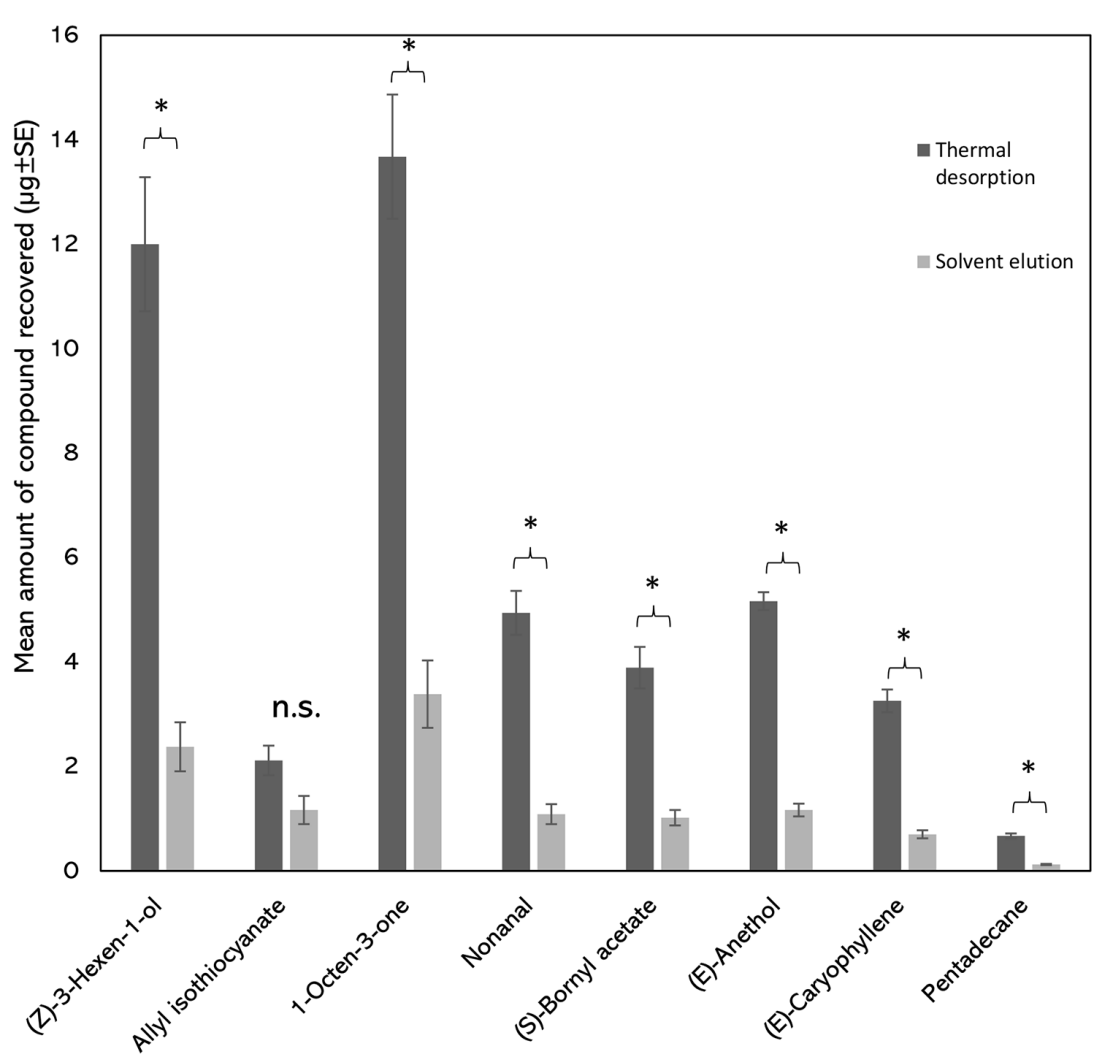
The effect of sample elution on compound recovery within the eight-component blend (mean µg ± standard error of mean (SEM). The length of PDMS tubes was 5 cm, and each experiment was conducted in a glass chamber (12 cm diam. × 6 cm height) across four replicates at 20 °C. PDMS tubes were either analysed using thermal desorption (dark grey) or solvent elution (light grey). Columns with an asterisk within one compound are significantly different at α = 0.001, Student’s t-test. n.s.=not significantly different

To determine the effect of desorption method on compound recovery, analytes from the PDMS tubes were either removed using thermal desorption or solvent elution with diethyl ether. Significantly greater amounts were recovered through thermal desorption compared to solvent elution for seven of eight compounds in the synthetic blend (t-test, d.f.= 6, *p* < 0.0001) (Fig. 1), which all had recovery rates of approximately 20% of those by thermal desorption. There was, however, no significant difference in the recovery of allyl isothiocyanate from the blend headspace between the two methods (*p* = 0.066). All eight compounds could be reliably recovered after 1 h of headspace sampling using 1 mL of ether to elute the PDMS tubes, therefore solvent elution was the chosen compound recovery method for the rest of the study. As discussed in more detail in the introduction, solvent extracts could be re-used for biological analysis of headspace samples, for example, using electrophysiological and behavioural assays.

### Experiment 2

The dosage of compounds dispensed into the headspace influenced compound recovery (Table 3) (Supplementary Fig. 3). All compounds could be retrieved from the headspace at 1, 10 and 100 µg applications. The range of recovery for the doses were: 5.19-69 ng from a 1-µg application, 144–561 ng from a 10-µg application, and 184–5781 ng from a 100-µg application. Based on these findings, 100 µg was selected as the dose for the following experiments, as this dose enabled recovery of compounds without overloading the GC. Moreover, as different parameters (e.g., tube length and sampling in open systems) were being investigated, which would likely reduce compound recovery in the headspace, this dose would increase chances of all compounds being captured.

**Table 3:** The effect of applied dose (1 µg, 10 or 100 µg) on the recovery of synthetic compounds (mean ng ± (SEM)). The length of PDMS tubes was 5 cm, and each experiment was conducted in a glass chamber (12 cm diam. × 6 cm height) across four replicates at 20 °C. KI = Kováts retention index

		Mean recovery of compound (ng ± SEM)		
KI	Compound	1 µg	10 µg	100 µg
844	(Z)-3-Hexen-1-ol	5.19 (± 0.87)	248.62 (± 28.65)	3951.03 (± 397.75)
854	Allyl isothiocyanate	12.24 (± 0.76)	166.94 (± 23.18)	1770.32 (± 279.07)
960	1-Octen-3-one	34.85 (± 8.27)	471.92 (± 51.93)	5781.58 (± 750.67)
1084	Nonanal	17.27 (± 3.4)	259.23 (± 22.40)	1890.45 (± 233.92)
1265	(*S*)-Bornyl acetate	32.05 (± 5.80)	496.57 (± 46.30)	1531.23 (± 203.02)
1275	(*E*)-Anethol	34.48 (± 7.58)	510.86 (± 40.32)	1325.55 (± 175.527)
1429	(*E*)-Caryophyllene	59.25 (± 9,70)	561.28 (± 33.02)	801.64 (± 106.51)
1500	Pentadecane	33.13 (± 3.48)	144.36 (± 5.9)	184.77 (± 18.17)

### Experiment 3

**Fig. 2:**
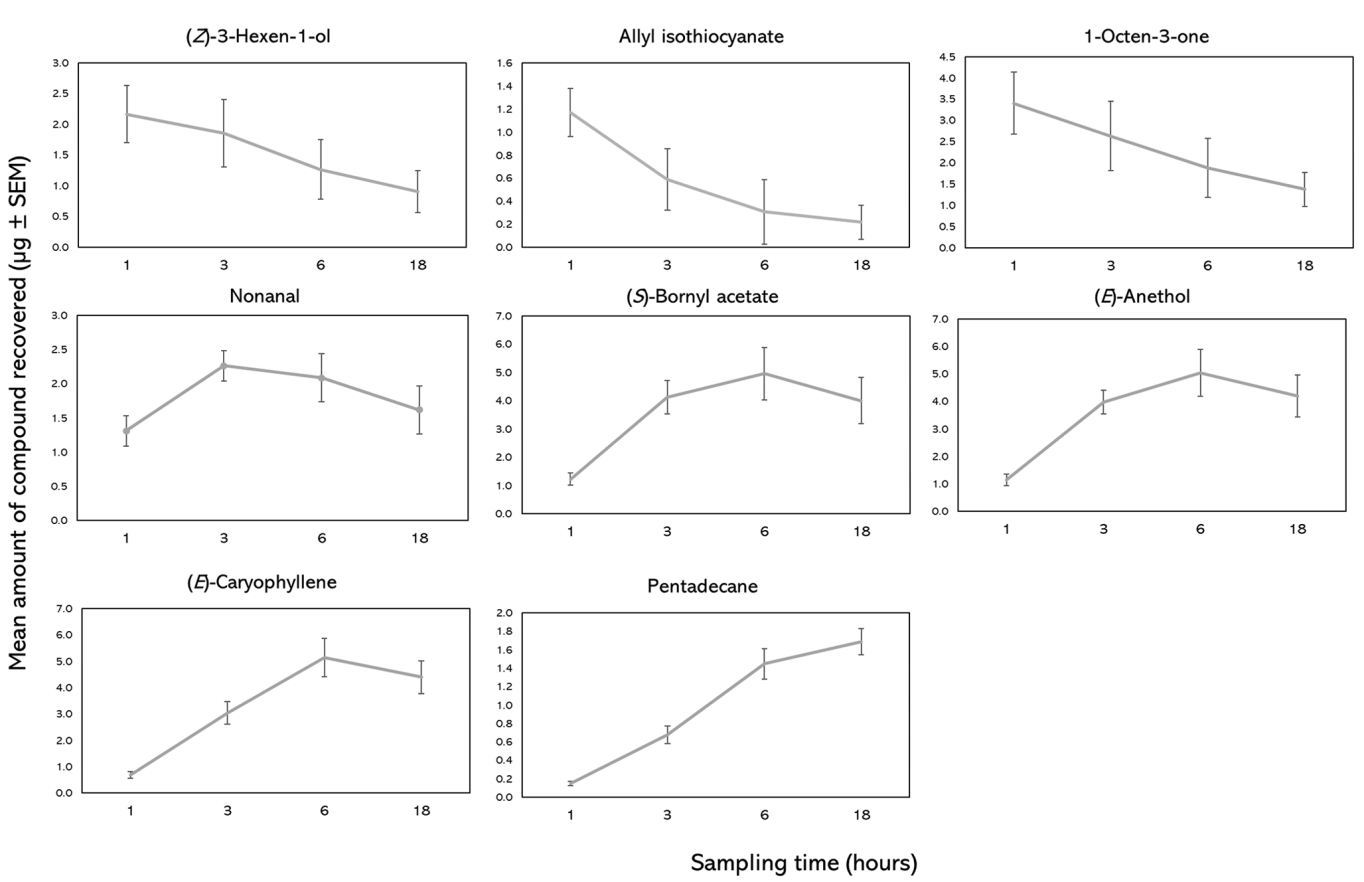
Change in recovery of compounds sampled over time (1 h, 3 h, 6 and 18 h) (mean µg ± SEM). The length of PDMS tubes was 5 cm, and each experiment was conducted in a glass chamber (12 cm diam. × 6 cm height) across four replicates at 20 °C

**Fig. 3:**
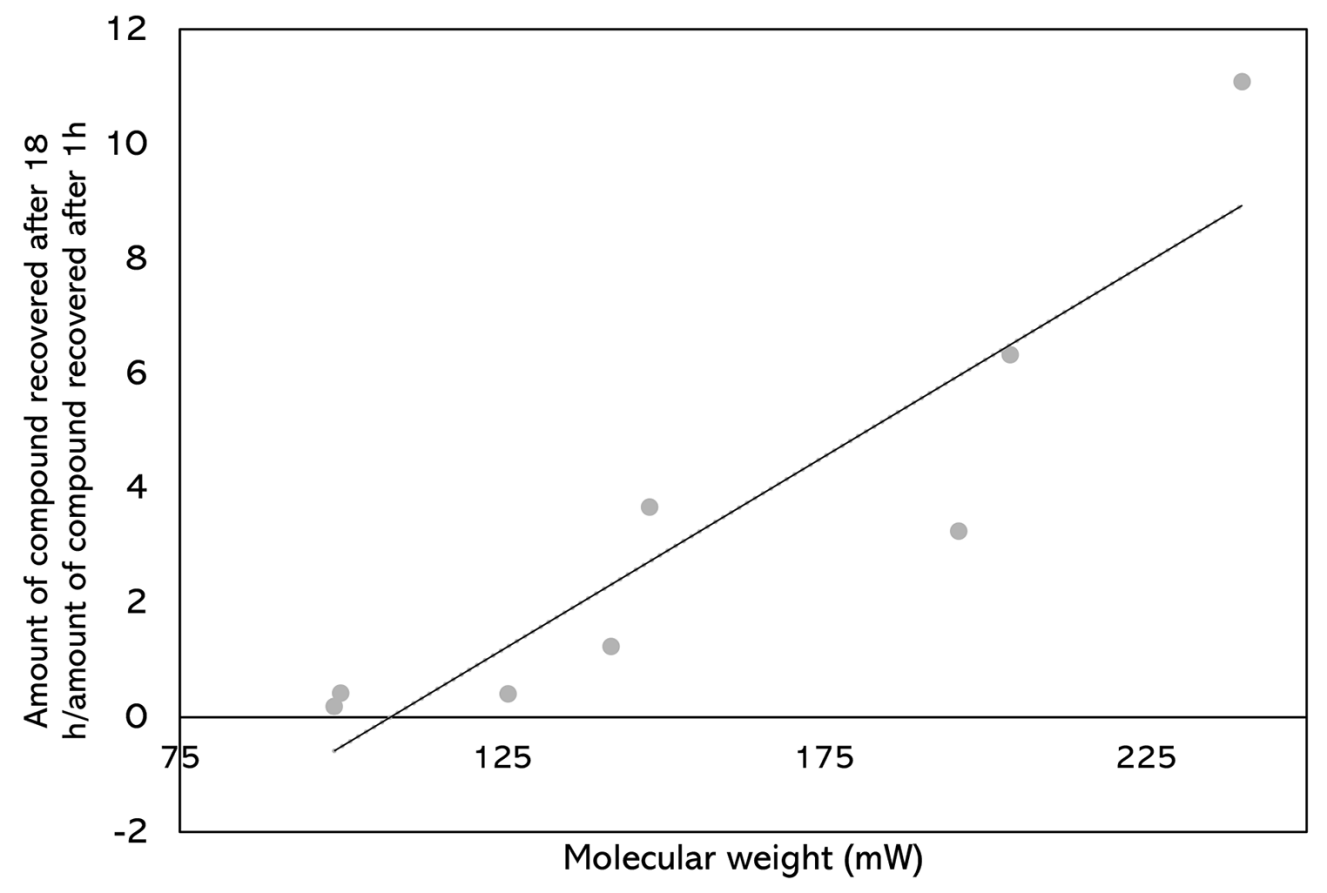
Correlation between molecular weight of compounds and ratio of recovery between 1 and 18 h of sampling

Having established in experiment 1 that solvent elution with 1 mL of ether could successfully recover all the components of the synthetic blend after 1 h of sampling from the headspace, collections were then performed for different lengths of time (1 h, 3 h, 6 and 18 h). For (*Z*)-hexen-1-ol, allyl isothiocyanate and 1-octen-3-one, recovery of compounds declined over time (Fig. 2). Contrastingly, for pentadecane, the recovery increased as sampling time increased. For the remaining compounds, an initial increase was observed between 1 and 3 h of sampling, then for nonane, a decrease between 3 and 18 h was observed. (*S*)-Bornyl acetate and (*E*)-anethol showed similar trends, demonstrating increases up in recovery between 1 and 6 h, followed by a decrease at 18 h. When plotting the recovery ratio of compounds (quantity of compound recovered after 18 h/quantity of compound recovered after 1 h, plotted against molecular weight of compound), there is a significant positive correlation between molecular weight and recovery ratio (Spearman’s rank, *r* = 0.952, *p* < 0.001, *n* = 8) (Fig. 3), suggesting that the greater the molecular weight of compounds, the higher the recovery of compounds at 18 h compared to 1 h.

### Experiment 4

**Fig. 4:**
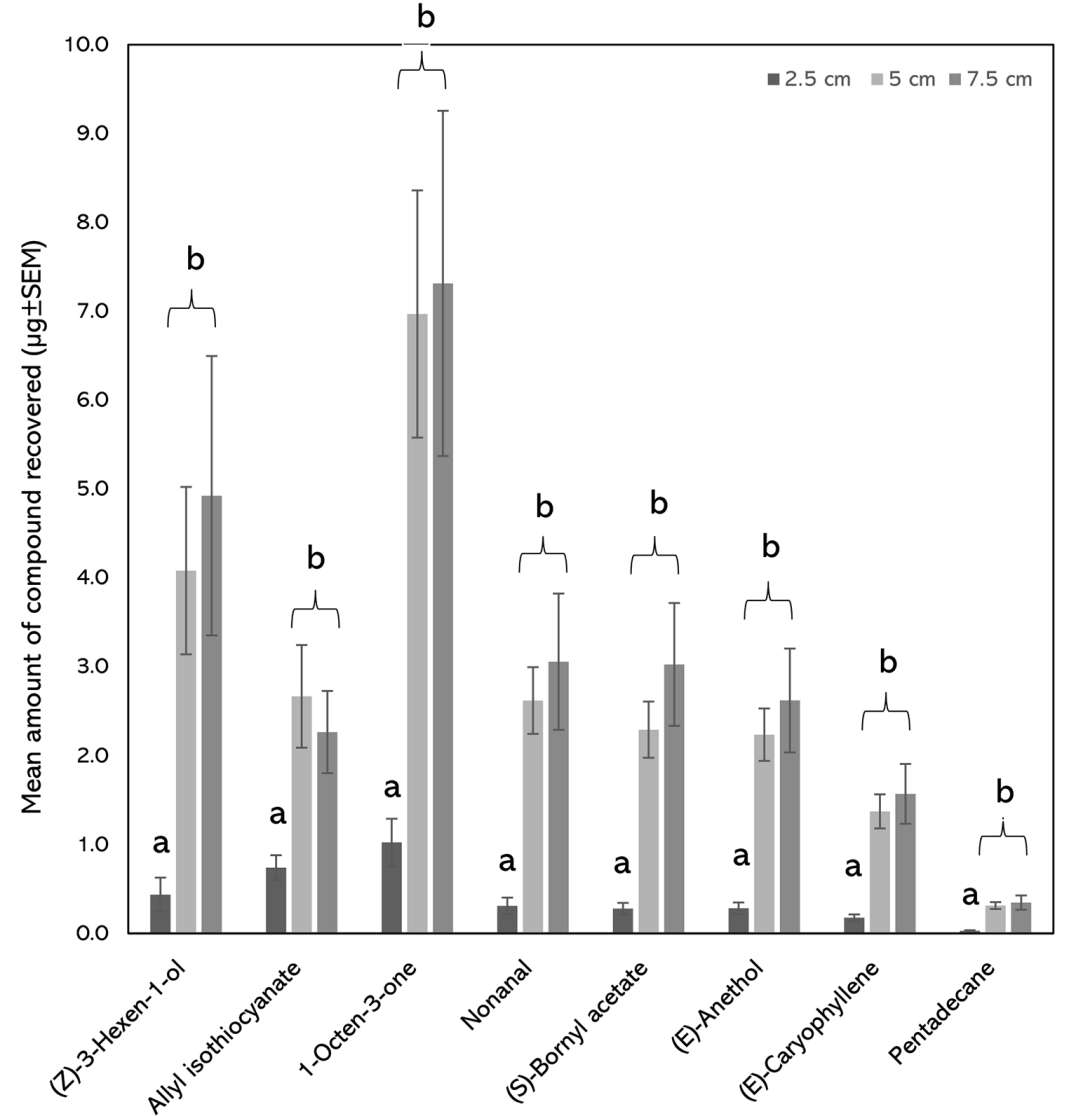
The effect of tube length on the recovery of compounds within the eight-component blend (mean µg ± SEM). The length of PDMS tubes was 2.5, 5 or 7.5 cm, and each experiment was conducted in a glass chamber (12 cm diam. × 6 cm height) across four replicates at 20 °C. Columns which share the same letter within one compound are not significantly different at α = 0.05, ANOVA.

To determine the influence of the length of PDMS tubing (i.e. absorptive volume) on compound recovery, tubes of three different lengths were exposed to the synthetic blend in the headspace of the glass chamber. For all eight compounds in the blend, there were significant differences in the amounts recovered across different tube lengths (*p* < 0.001), although no significant differences in recovery were observed when comparing 5 or 7.5 cm tubes (*p* > 0.05) (Fig. 4).

### Experiment 5

To determine whether compounds can be recovered from PDMS tubes using solvent elution in an open system, three conditions were compared: (a) closed system, (b) open system with PDMS tube 4 cm from source and (c) open system with PDMS tube 1 cm from source. No compounds could be detected from PDMS tubes suspended 4 cm above the filter disc, whereas six of the eight compounds were detectable when the PDMS tube was suspended 1 cm from the source in an open system (Table 4). Recovery of compounds from 1 cm distance were in the range of 0.04-0.19 µg (Table 4). A dose response experiment under open conditions demonstrated that recovery of five out of eight compounds is achievable at 100 µg and 10 µg doses, and four compounds at a 1 µg dose (Table 5).

**Table 4:** The effect of open versus closed system on the recovery of synthetic compounds (mean µg ± SEM). The dose applied to the filter paper was 100 µg. The length of PDMS tubes was 5 cm, and each experiment was either conducted in a glass chamber (12 cm diam. × 6 cm height) across four replicates at 20 °C, or in an open system under the same conditions

	Mean recovery of compound (µg ± SEM)		
Compound	Closed system	Open system (4 cm from source)	Open system (1 cm from source)
(*Z*)-3-Hexen-1-ol	2.17 (± 0.42)	0.0	0.0
Allyl isothiocyanate	1.57 (± 0.35)	0.0	0.0
1-Octen-3-one	3.59 (± 0.70)	0.0	0.040 (± 0.016)
Nonanal	1.51 (± 0.23)	0.0	0.087 (± 0.031)
(*S*)-Bornyl acetate	1.33 (± 0.15)	0.0	0.18 (± 0.062)
(*E*)-Anethol	1.25 (± 0.21)	0.0	0.19 (± 0.074)
(*E*)-Caryophyllene	7.60 (± 0.14)	0.0	0.088 (± 0.038)
Pentadecane	1.82 (± 0.311)	0.0	0.047 (± 0.017)

**Table 5:** The effect of dose in open systems on the recovery of synthetic compounds (mean ng ± SEM). The length of PDMS tubes was 5 cm, sampling distance at 1 cm, and each experiment was performed at 20 °C

	Mean recovery of compound (ng ± SEM)		
Compound	100 µg	10 µg	1 µg
(*Z*)-3-Hexen-1-ol	0.0	0.0	0.0
Allyl isothiocyanate	0.0	0.0	0.0
1-Octen-3-one	74.07 (± 22.14)	4.79 (± 0.28)	0.0
Nonanal	45.07 (± 9.98)	4.80 (± 0.32)	0.0
(*S*)-Bornyl acetate	46.49 (± 17.21)	9.06 (± 2.08)	1.85 (± 0.14)
(*E*)-Anethol	55.59 (± 18.48)	12.75 (± 2.15)	2.20 (± 0.21)
(*E*)-Caryophyllene	27.48 (± 5.36)	16.87 (± 6.73)	1.74 (± 0.17)
Pentadecane	10.54 (± 3.15)	9.54 (± 1.49)	3.20 (± 0.24)

### Experiment 6

**Fig. 5:**
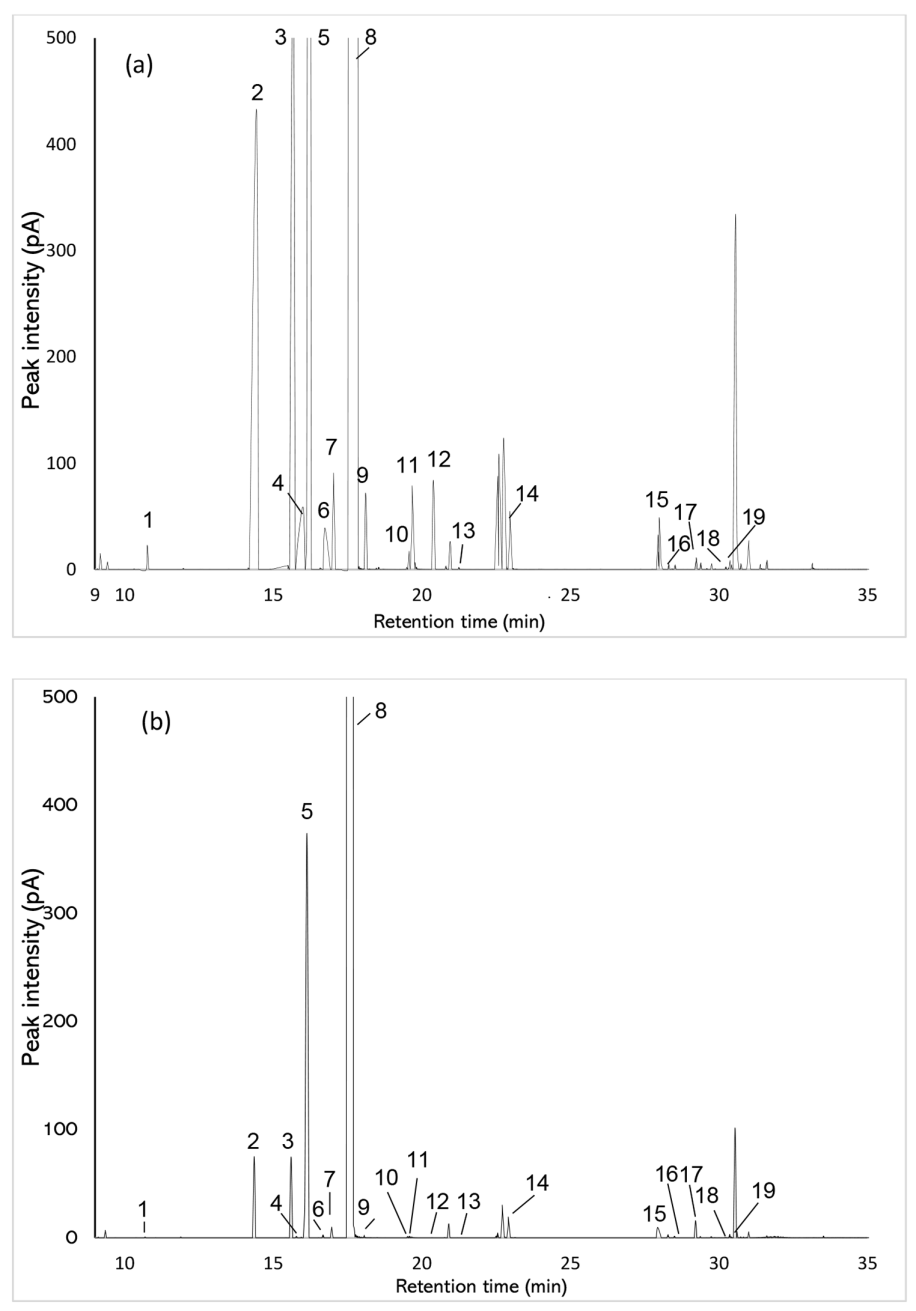
Representative GC trace of orange (*Citrus sinensis*) headspace sampled by **(a)** dynamic headspace collection (air entrainment) and **(b)** PDMS tubing. The length of PDMS tubes was 5 cm, and each experiment was conducted in a glass chamber (12 cm diam. × 10 cm height) across four replicates at 20 °C. The FID peaks were identified as: (1) (*E*)-2-hexenal, (2) α-pinene, (3) sabinene, (4) β-pinene, (5) myrcene, (6) α-phellandrene, (7) 3-carene, (8) limonene, (9) (*E*)-ocimene, (10) nonanal, (11) terpinolene, (12) (*E*)-4,8-dimethyl-1,3,7-nonatriene [(*E*)-DMNT], (13) citronellal, (14) decanal, (15) hexyl hexanoate, (16) copaene, (17) (*E*)-caryophyllene, (18) humulene, (19) γ-selinene. Zoomed-in, representative GC traces are presented in Fig. 5, as the dominant peak (limonene) makes it difficult to see compounds lower in abundance when observing the full trace, although full traces are illustrated in Supplementary Fig. 4

An experiment was established to investigate whether VOCs could be sampled from the headspace of oranges. Under open systems, VOCs produced by oranges could not be captured, therefore a closed system within a glass chamber was established. There were no qualitative differences in the compounds captured when comparing PDMS tubing with dynamic headspace collections, (Fig. 5; Table 5); however, dynamic headspace collections captured greater quantities of compounds (Table 6). Zoomed-in, representative GC traces are presented in Fig. 5, as the dominant peak (limonene) makes it difficult to see compounds lower in abundance when observing the full trace, although full traces are illustrated in Supplementary Fig. 4.

**Table 6:** Compounds identified from the headspace of oranges using air entrainment and PDMS tubing (mean ng ± SEM). The length of PDMS tubes was 5 cm, and each experiment was conducted in a glass chamber (12 cm diam. × 10 cm height) across four replicates at 20 °C for 1 h. *tentative identifications by comparison of GC retention indices (KI values) and mass spectra with those of authentic standards. Tentative identity of compounds not marked with an asterisk was confirmed by GC peak enhancement via co-injection with authentic standards

				Air entrainment		PDMS	
Peak no.	Tentative ID	KI	Exp. KI	Mean	SEM	Mean	SEM
1	(*E*)-2-Hexenal	831	830	**280.78**	34.03	**2.99**	0.45
2	α-Pinene	937	937	**5452.50**	110.43	**141.02**	33.73
3	Sabinene	971	971	**9013.17**	484.54	**269.75**	100.96
4	β-Pinene	976	976	**409.93**	9.89	**13.56**	4.66
5	Myrcene	986	985	**32357.23**	4120.93	**520.49**	132.95
6	α-Phellandrene*	999	999	**719.67**	73.73	**14.68**	3.23
7	3-Carene	1008	1008	**2967.52**	430.23	**57.12**	17.80
8	Limonene	1034	1033	**1550567.40**	193256.27	**36551.47**	6542.52
9	(*E*)-Ocimene	1042	1041	**1719.67**	786.58	**13.54**	5.08
10	Nonanal	1083	1084	**712.23**	99.75	**7.71**	2.22
11	Terpinolene*	1086	1086	**1986.51**	871.97	**10.56**	3.79
12	(*E*)-DMNT	1106	1106	**1524.26**	529.01	**3.89**	1.41
13	Citronellal*	1133	1134	**106.74**	50.83	**1.32**	0.06
14	Decanal	1185	1186	**1686.34**	518.56	**26.90**	3.60
15	Hexyl hexanoate*	1371	1371	**2219.16**	710.83	**29.74**	12.60
16	Copaene*	1391	1388	**118.89**	44.94	**1.58**	0.14
17	(*E*)-Caryophyllene	1432	1434	**630.84**	155.56	**24.72**	3.12
18	Humulene	1466	1468	**57.23**	19.53	**1.68**	0.06
19	γ -Selinene*	1492	1494	**310.35**	76.45	**6.04**	0.45

## Discussion

To understand the role of VOCs as semiochemicals mediating interactions between organisms, it is crucial to extract them from biological systems such that the extract can then be used for repeated chemical and behavioural analysis. Here, we demonstrate that solvent elution of short PDMS pieces extracts VOC semiochemicals from closed and open headspaces and that parameters, including tube length and sampling time, can significantly influence compound recovery.

A comparison of thermal desorption versus solvent elution for headspace VOC sampling demonstrated significantly greater recovery of constituents of an eight-component synthetic blend with thermal desorption. This is unsurprising, as thermal desorption causes all absorbed compounds to be removed from the tube, enabling whole-sample analysis. Contrastingly, with solvent elution, a fixed amount of organic solvent is used to elute the tubing, and a portion of the total sample can be analysed using GC-FID/GC-MS. Due to the simple handling and low cost of PDMS tubing compared to SPME (Kallenbach et al. 2015), the two different analysis methods could be applied concurrently when sampling headspace, with one piece of PDMS tubing analysed by thermal desorption, ensuring increased analytical sensitivity of the headspace, and the second tube for solvent elution, which could then be used for repeated analytical and biological assays. This highlights an advantage over other VOC sampling techniques, as several PDMS tubes can be used simultaneously across biological or technical replicates and stored until further analysis.

PDMS tubing can be cut into pieces of different sizes, which can subsequently influence sampling efficiency, highlighting the versatility of the technique. Whilst most studies to date adopt thermal desorption for PDMS sample analysis, where silicone tubes are cut into a particular size (often 5 mm) (Kong et al. 2021; Song et al. 2022a, b; Lammers et al. 2021; Lee Díaz et al. 2022) to fit into a Tenax tube, here we demonstrate the length of tubing can in fact influence the recovery of compounds. A significant increase in compound recovery was observed when 2.5-5 cm PDMS tubing was used for sampling, although not between 5 and 7.5 cm tubes. This may indicate that the tube was becoming saturated, although tests using longer tube lengths would be required to confirm this. It could also indicate that the majority of the analyte vapor in the headspace was collected with the 5 cm tube, and therefore increasing the tube length to 7.5 cm had little influence on the quantity of compounds recovered. Another important consideration of this work is that the length of sampling time can also have an influence on recovery of compounds. This has been shown previously, where plateauing of compound recovery was observed as sampling time increased, suggesting equilibrium had been reached, and for certain compounds, declines in the quantity recovered were observed over time (Alborn et al. 2021; Augusto and Luiz Pires Valente 2002; Jeleń et al. 2000; Šanda et al. 2012; Song et al. 1997). This corroborates our findings, showing declines in the recovery of (*Z*)-3-hexenol, allyl isothiocyanate and 1-octen-3-one over the course of the experiments. The lower molecular weight compounds in the synthetic blend show a decline in recovery with increasing sampling times, whereas higher molecular weight compounds generally show either no difference or increased recovery as sampling time increases. These heavier compounds evaporate more slowly, meaning equilibrium is reached over a longer time, whereas the lower molecular weight compounds evaporate more rapidly. This has been observed previously with SPME headspace sampling, whereby lower molecular weight compounds from apples equilibrated with the fibre within 5 min of sampling, whereas higher molecular weight compounds evaporated more slowly and therefore took longer to equilibrate (Matich et al. 1996).Taken together, these findings support the observation that absorption and headspace equilibration process differs for different compounds, which should be considered when drawing conclusions from biological headspace collections (Song et al. 1997).

As well as using the tubing to capture from the headspace of an eight-component synthetic blend, we have also demonstrated that PDMS tubing can collect VOCs from the headspace of plant material within an enclosed system, demonstrating its suitability to capture naturally occurring VOCs from biological sources. Of the total amount of compounds extracted using dynamic headspace collection, all compounds representing a range of chemical classes (aldehydes, ketones, alcohols, mono- and sesquiterpenes) were successfully captured by PDMS tubing, many of which were previously reported from orange headspace (Chamberlain et al. 2012; Hou et al. 2020; Cuevas et al. 2017; Centonze et al. 2019; Fancelli et al. 2018). However, as the orange extracts collected using dynamic headspace collection overloaded the GC column when concentrated down to 100 µL, and were therefore not concentrated down before GC analysis, we cannot conclude that no qualitative differences in the production of less abundant compounds are observed when these extracts are more concentrated. Moreover, it should be noted that oranges were used here as a model species, so we cannot conclude that no qualitative differences in volatile recovery would be observed for other plant species, which would require further experimentation. Unsurprisingly, quantities captured using solvent elution of PDMS tubing are substantially lower relative to dynamic headspace collection, as the latter requires the constant flow of charcoal-purified air over the headspace, increasing the accumulation of compounds on the filter. Although highly likely, it remains to be demonstrated by bioassays (e.g. electrophysiology, behaviour) that the extracts resulting from solvent elution of PDMS contain semiochemicals at physiologically relevant quantities. Collection of VOCs from the open system above the headspace of oranges was unsuccessful, with only one replicate of four showing capture of certain VOCs, contrary to what was demonstrated in Experiment 5. However, this may be expected, because the synthetic blend was applied to a point source (filter paper) in experiment 5.

**Fig. 6:**
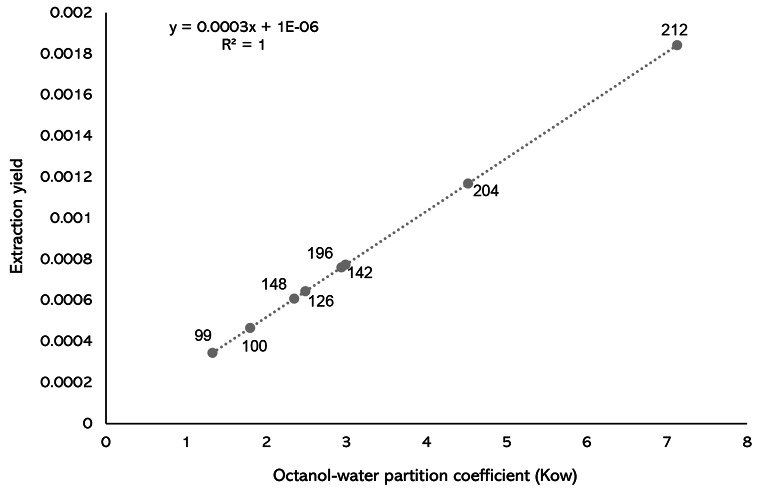
Relationship between molecular weight, extraction yield and octanol-water partition coefficient (K_ow_) for each of the synthetic compounds within the eight-component blend. Compound names belonging to molecular weight are as follows: (*Z*)-3-Hexen-1-ol: 99, Allyl isothiocyanate: 100, (*S*)-Bornyl acetate: 148, 1-Octen-3-one: 126, (*E*)-anethol: 196; Nonanal: 142, (*E*)-Caryophyllene: 204, Pentadecane: 212

The recovery of compounds from the headspace depends on the partition coefficient of the analyte between the PDMS and the sample headspace and the partition coefficient between the PDMS and the eluting solvent. Analyte recovery is further influenced by the ability of compounds to diffuse into the inner surface of PDMS tubing. On the outer surface of the PDMS tube, the concentration of analyte is greater than on the inner surface of the tube; therefore, the concentration of analyte recovered during solvent elution is dependent on the ability of the compound to dissolve into the polymer and onto the inner tube surface. This is dependent on the octanol-water partition coefficient (K*ow*) of the analyte molecules; the higher the K*ow*, the greater the lipophilicity of the compound, and therefore the more that can dissolve into the PDMS tubing. The extraction yield of each compound shows a linear relationship with K*ow*, whereby a higher K*ow* means a higher analyte concentration within the PDMS inner surface. Extraction yield is not, however, directly proportional to molecular weight (Fig. 6).

Our results show extraction of short pieces of PDMS tubing with small amounts of diethyl ether solvent captures VOCs from the headspace of biological samples, and the resulting extracts can be used for repeated chemical analyses. Length of PDMS tubing and length of sampling time need to be considered before sampling, because they significantly influence the accumulation of analytes in the polymer. Also, based on Vuts et al. (2020), increasing eluent volume will increase the extracted amount of analytes. Another advantage of using the system described here is the relatively low cost of PDMS tubing.

### Electronic supplementary material

Below is the link to the electronic supplementary material.

Supplementary Material 1
