# Supplementary material for: Solvent Extraction of PDMS Tubing as a New Method for the Capture of Volatile Organic Compounds from Headspace
Source: J Chem Ecol. 2024 Jan 22;50(3-4):85–99. doi: 10.1007/s10886-024-01469-y (PMC11041800; doi:10.1007/s10886-024-01469-y)
Supplement: Supplementary file 1 — Supplementary Material 1 [file 10886_2024_1469_MOESM1_ESM.docx]

**Supplementary information**

**
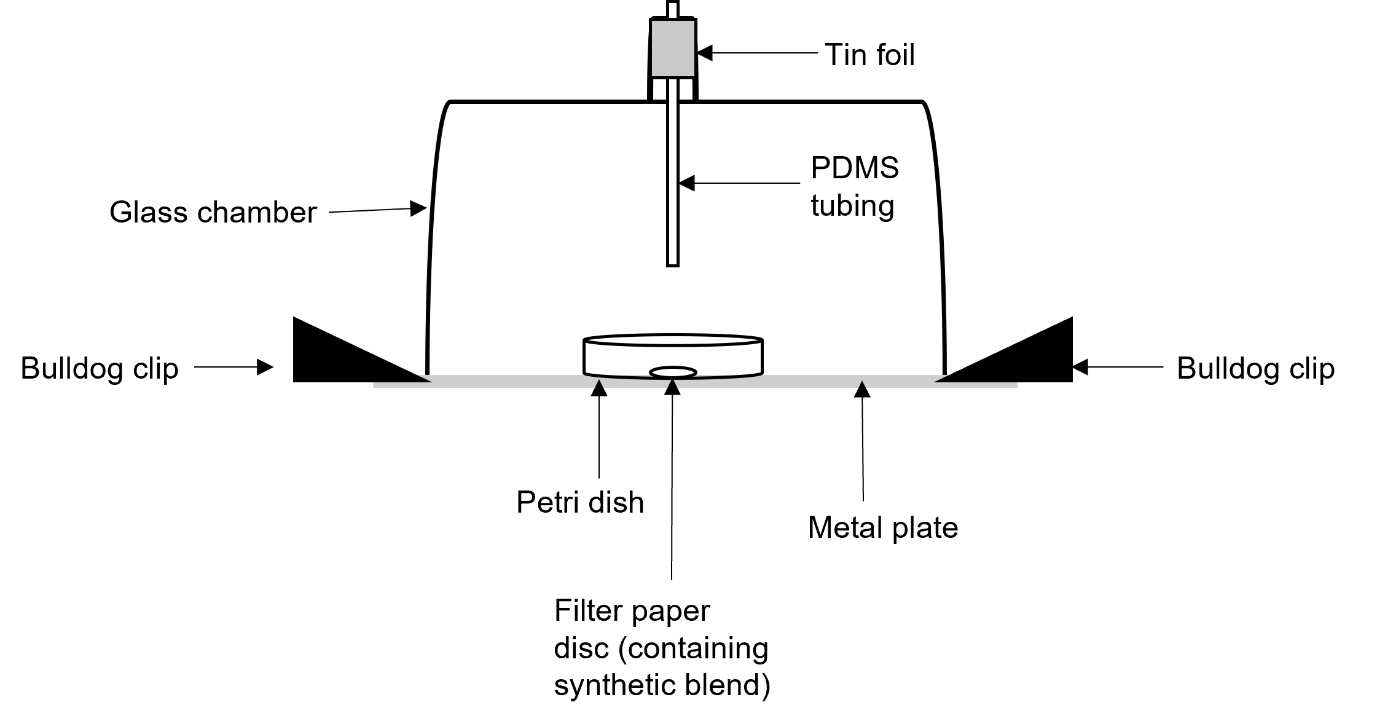
**

Supplementary Figure 1 | Set-up of headspace PDMS sampling, of synthetic blend within glass chamber.


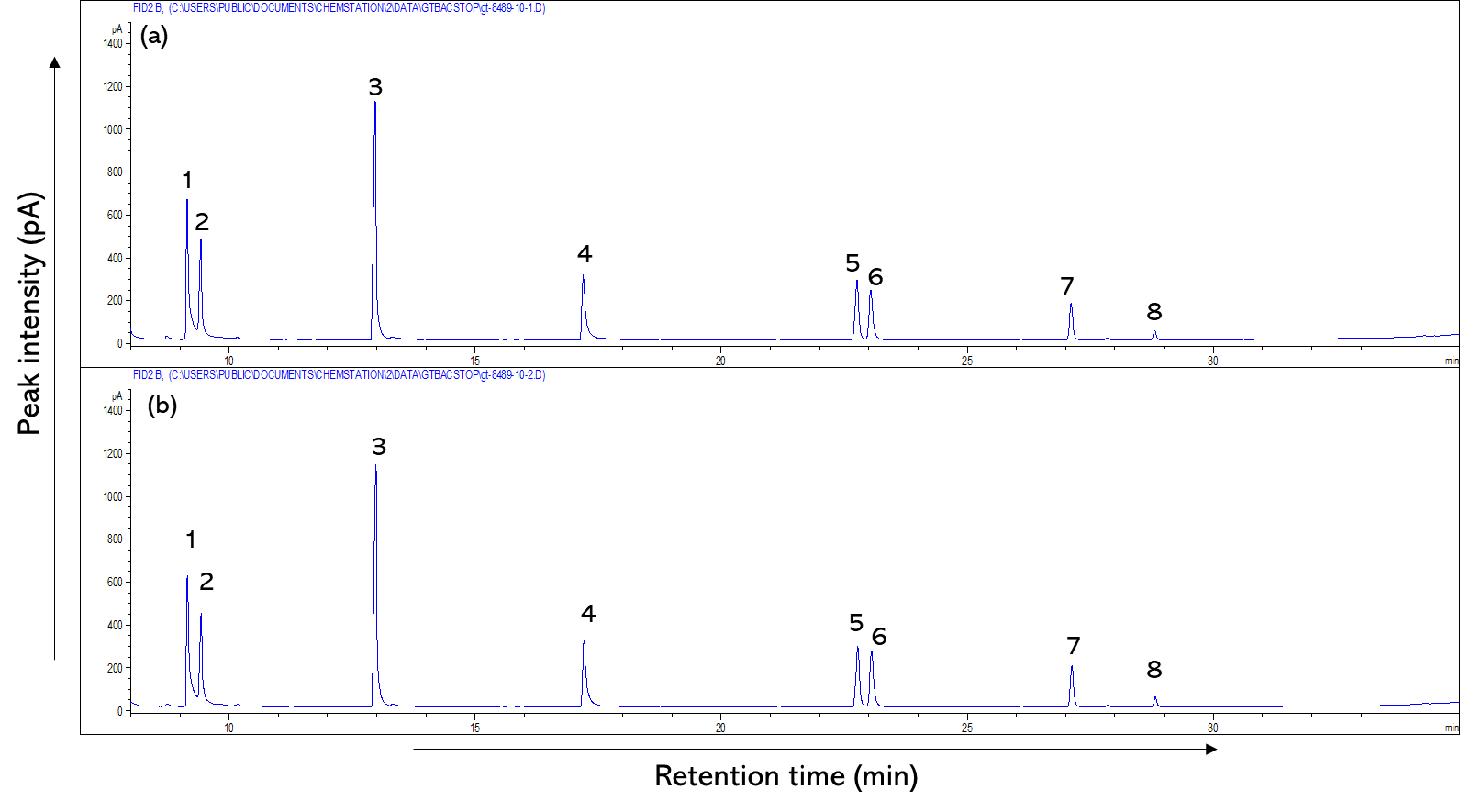


Supplementary Figure 2 | Representative GC analysis (on a HP-1 column) of VOCs collected from PDMS tubes which were either (a) sealed at the top with a crocodile clip or (b) unsealed. Peak numbers correspond to: (1) (*Z*)-3-hexen-1-ol, (2) allyl isothiocyanate, (3) 1-octen-3-one, (4) nonanal, (5) (*S*)-bornyl acetate, (6) (*E*)-anethol, (7) (*E*)-caryophyllene, (8) pentadecane.

**
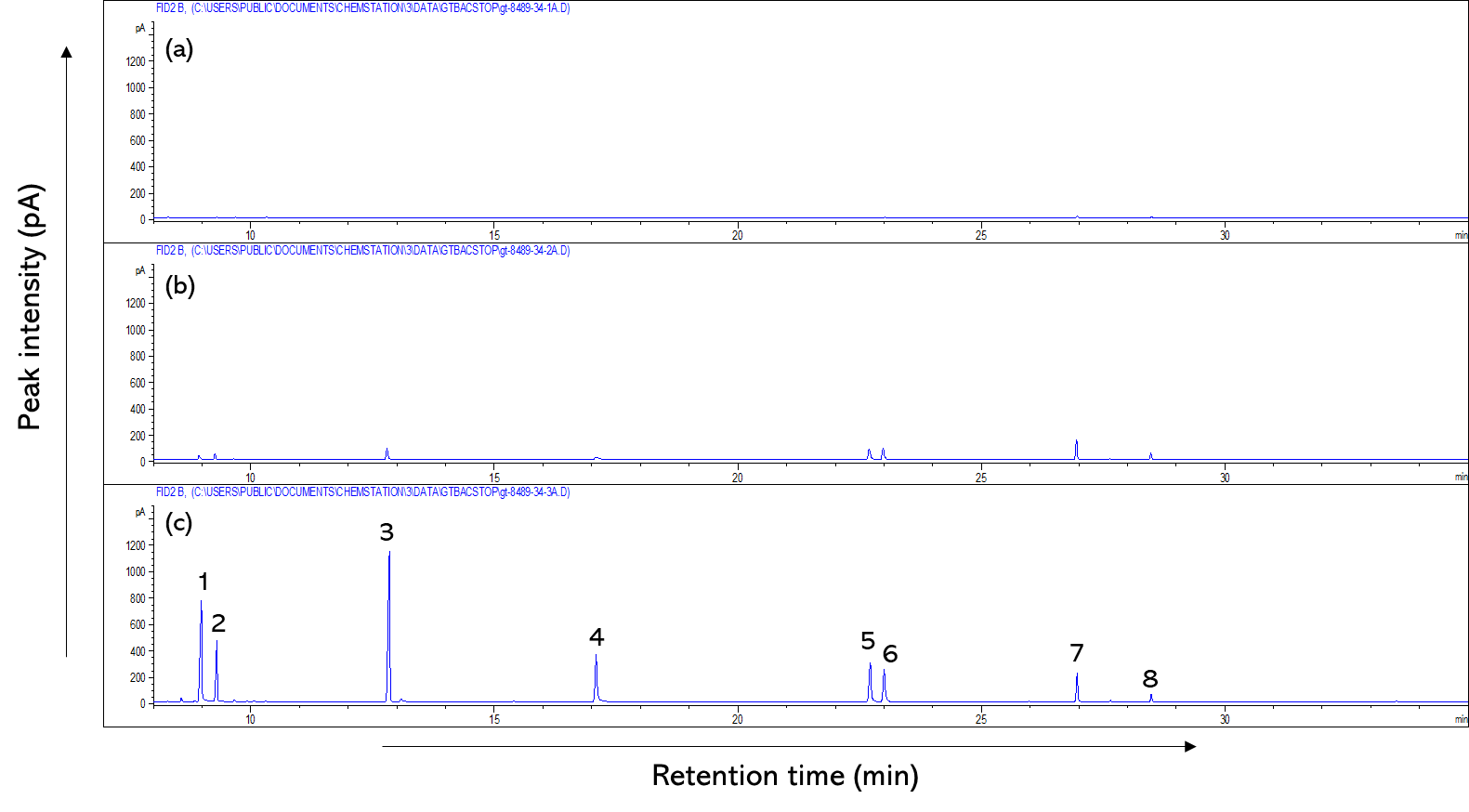
**

Supplementary Figure 3 | Representative GC analysis (on a HP-1 column) of VOCs from PDMS tubes exposed to 10 µL of the synthetic blend at (a) 1 µg, (b) 10 µg and (c) 100 µg. Peak numbers correspond to: (1) (*Z*)-3-hexen-1-ol, (2) allyl isothiocyanate, (3) 1-octen-3-one, (4) nonanal, (5) (*S*)-bornyl acetate, (6) (*E*)-anethol, (7) (*E*)-caryophyllene, (8) pentadecane.


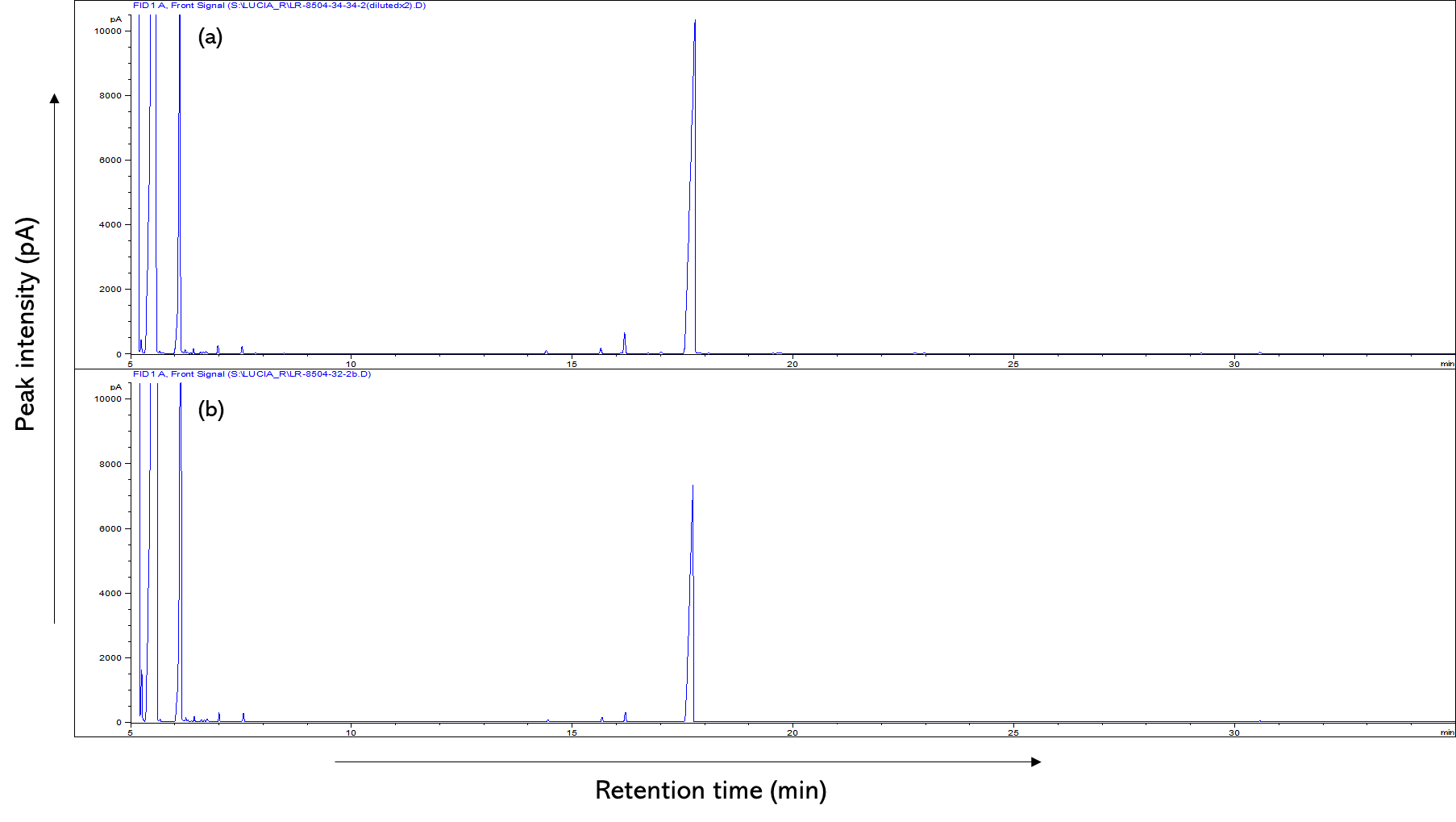


Supplementary Figure 4 | Representative GC analysis (on a HP-1 column) of VOCs collected from orange (*Citrus sinensis*) headspace, sampled by (a) dynamic headspace collection and (b) PDMS tubing. The length of PDMS tubes was 5 cm, and each experiment was conducted in a glass chamber (12 cm diam. × 10 cm height) across four replicates at 20°C.
